# Graphene Family Nanomaterials in Ocular Applications: Physicochemical Properties and Toxicity

**DOI:** 10.1021/acs.chemrestox.0c00340

**Published:** 2021-05-27

**Authors:** Sedigheh Borandeh, Vahid Alimardani, Samira Sadat Abolmaali, Jukka Seppälä

**Affiliations:** † Polymer Technology, School of Chemical Engineering, Aalto University, Kemistintie 1, 02150 Espoo, Finland; ‡ Department of Pharmaceutical Nanotechnology, School of Pharmacy, Shiraz University of Medical Sciences, 7146864685 Shiraz, Iran

## Abstract

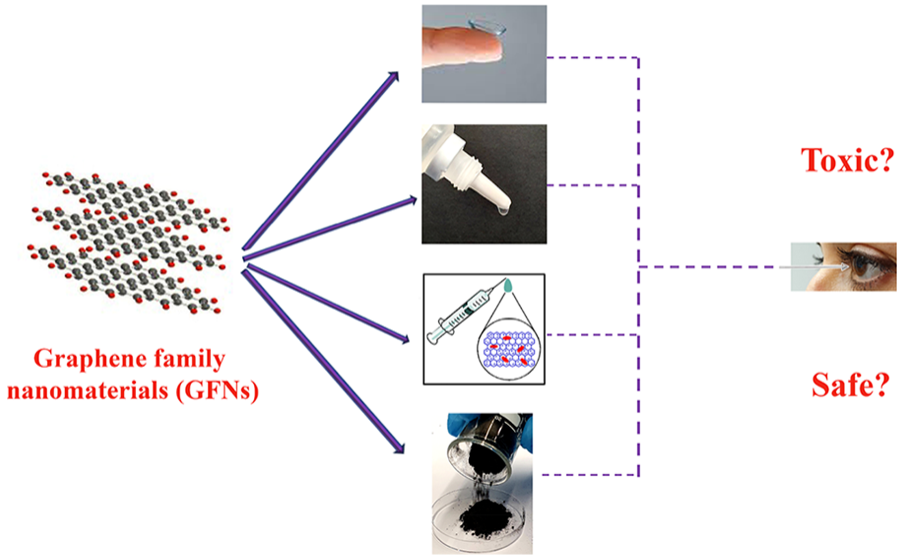

Graphene family nanomaterials (GFNs) are rapidly emerging for ocular applications due to their outstanding physicochemical properties. Since the eyes are very sensitive organs and the contact between the eyes and GFNs in eye drops, contact lenses, intraocular drug delivery systems and biosensors and even the workers handling these nanomaterials is inevitable, it is necessary to investigate their ocular toxicities and physiological interactions with cells as well as their toxicity mechanisms. The toxicity of GFNs can be extremely affected by their physicochemical properties, including composition, size, surface chemistry, and oxidation level as well as dose and the time of exposure. Up to now, there are several studies on the *in vitro* and *in vivo* toxicity of GFNs; however, a comprehensive review on ocular toxicity and applications of GFNs is missing, and a knowledge about the health risks of eye exposure to the GFNs is predominantly unspecified. This review highlights the ocular applications of GFNs and systematically covers the most recent advances of GFNs’ physicochemical properties, *in vitro* and *in vivo* ocular toxicity, and the possible toxicity mechanisms as well as provides some perspectives on the potential risks of GFNs in material development and biomedical applications.

## 1 Introduction

In the field of pharmaceutical and biotechnology sciences, nanotechnology is in high demand these days, leading to the desired products or therapeutic outcomes. Development of nanomaterials has increasingly assigned a large number of studies in academic and industrial groups to generate engineered advanced materials and biomedical systems.^1,2^ However, nanomaterials may lead to toxic effects on the cells and subcellular organelles owing to their nanosize and high surface area. There are several exposure ways to nanomaterials through inhalation (nose and lung) and contact (skin and eye). The personnel who are working with nanomaterials are at a high risk of exposure while they are preparing or handling nanomaterials. Therefore, due to continuously expanding demands of nanomaterials and increasing exposure to them, it is essential to evaluate the potential risks and hazards of nanomaterials from the human health and safety perspective.

**Figure 1:**
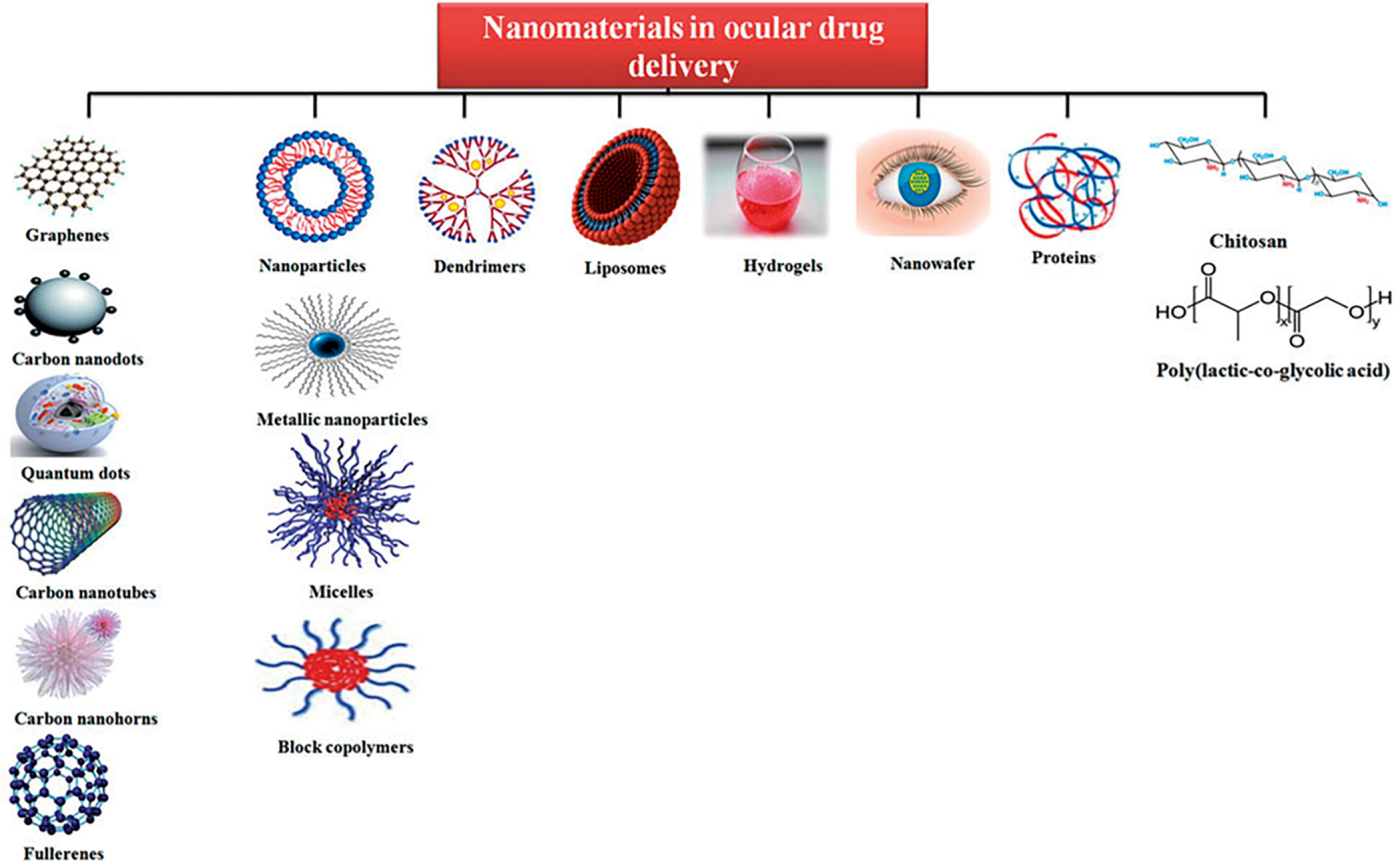
Different nanomaterials as ocular drug delivery systems. Reprinted with permission from ref (3). Copyright 2016 Taylor & Francis.

Among various human organs, investigation of nanomaterials’ toxicity on the eyes is crucially important due to high levels of eye exposure with nanomaterials during manufacturing, use, and disposal. Moreover, recently, there are several outstanding reports on the applications of nanomaterials in ocular applications such as ocular drug delivery, eye drops, and contact lenses.^3^ Design and development of novel therapy techniques using nanomaterials might lead to novel therapeutic methods in ophthalmology. Figure 1 presents different types of nanomaterials that have been used as ocular drug delivery systems.

**Figure 2:**
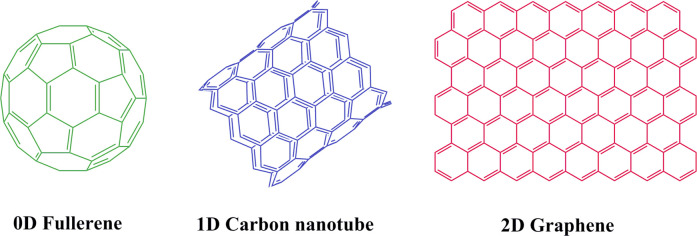
Structures of fullerene (0D), CNT (1D), and graphene (2D).

Among different nanomaterials, the carbonaceous materials, such as graphene, carbon nanotube (CNT), and fullerene (Figure 2) are at the forefront of advanced materials. Due to unique structure of carbon, it is able to form several allotropes, which result in a broad range of structures that exist in forms of zero-dimensional (0D) to three-dimensional with different shapes and properties from hard to soft, from insulative to semiconductive/conductive and from light absorbing to diaphanous.^4−6^

**Figure 3:**
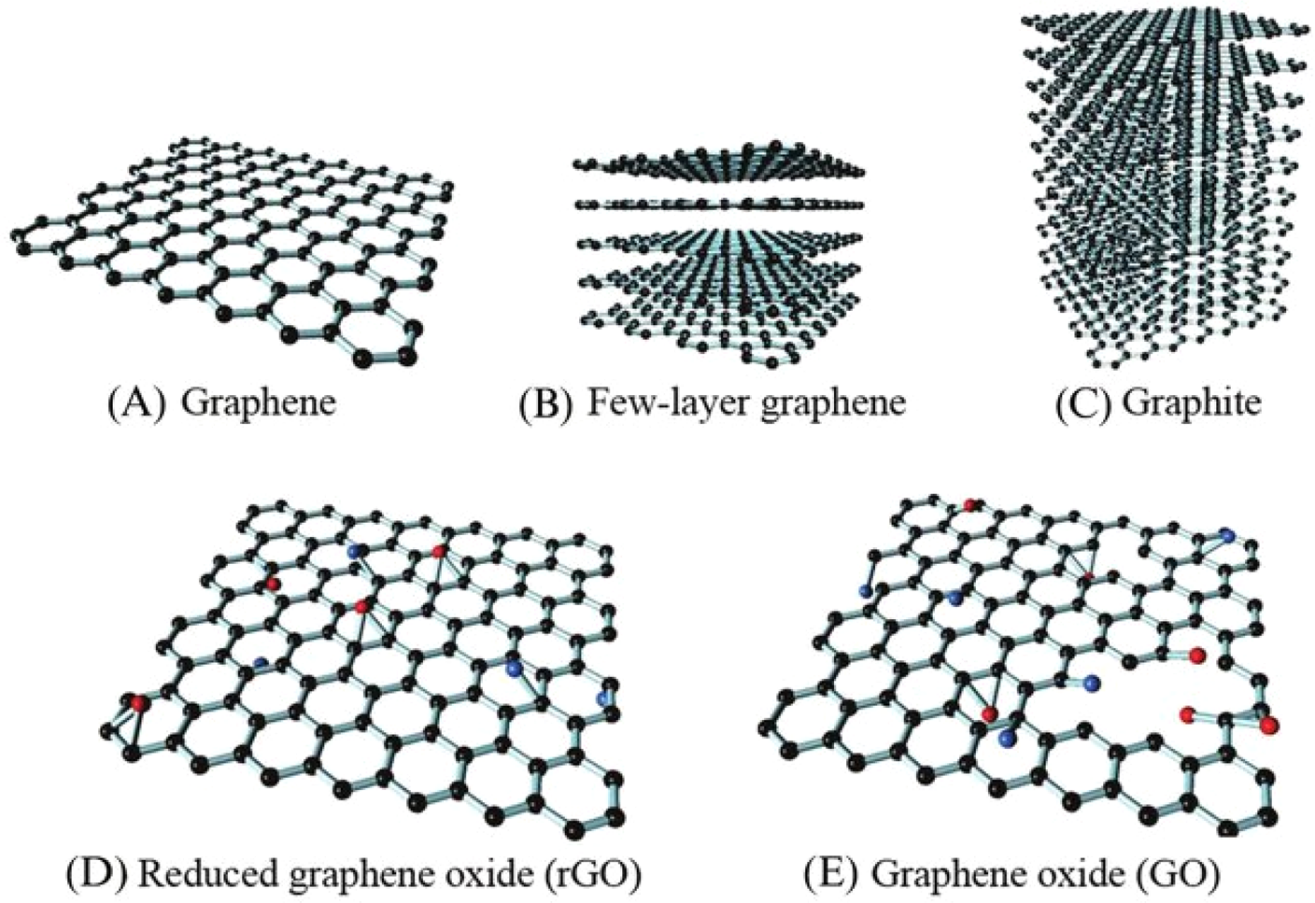
Most important GFN analogs. Reprinted with permission from ref (17). Copyright 2016 Elsevier.

In 2010, the Nobel Prize in Physics was awarded to Geim and co-workers for a 2D sheet-like material, graphene, which has shown the importance of transformative potential of carbon nanomaterials.^6^ Graphene has received tremendous interest from academia to industries owing to its outstanding physicochemical and structural properties.^7−11^ The 2D sheet of carbon atoms in a honeycombed network provides graphene with a large surface to volume ratio and high mechanical, thermal, and electrical properties.^12−14^ Generally, graphene, a single sheet of graphite that is held together by a backbone of overlapping sp^2^ hybrid bonds, is a part of graphene family nanomaterials (GFNs). Graphene, few-layer graphene (FLG, 2–10 layers), graphite, reduced graphene oxide (rGO), graphene oxide (GO), graphene nanoplatelets (GNP), and graphene quantum dots (GQDs) are the most important GFN analogs (Figure 3).^8,15,16^

**Figure 4:**
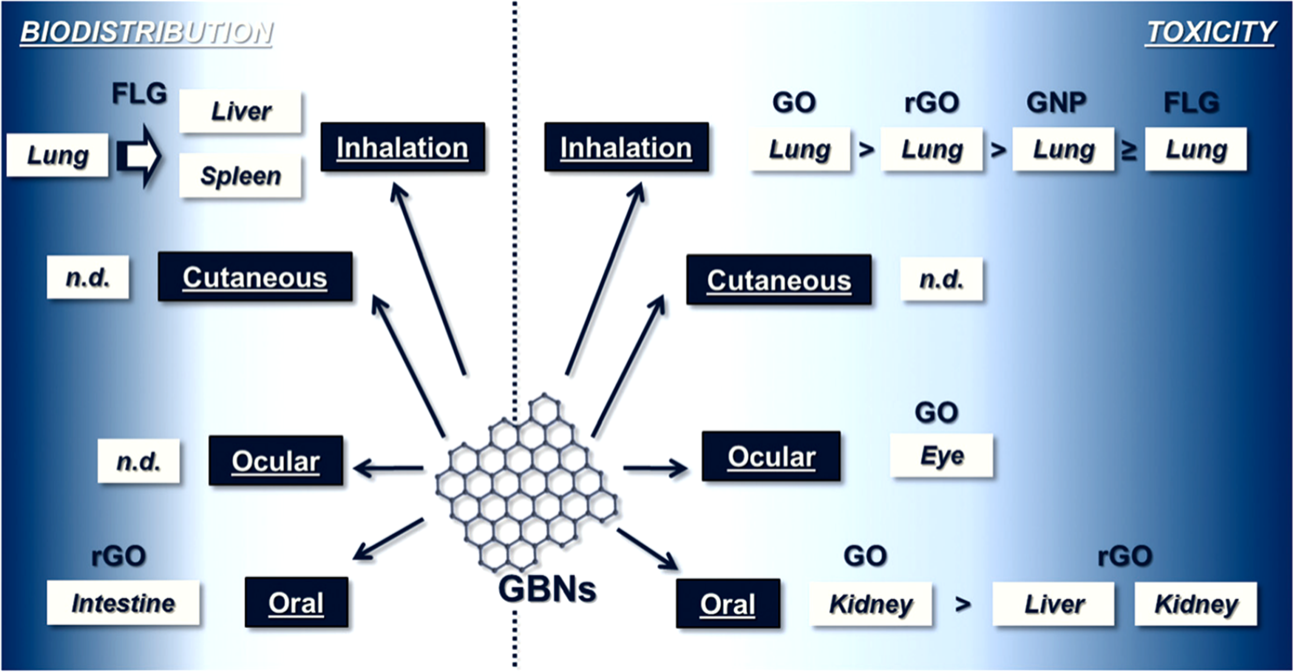
Summary of the existing knowledge on toxicity of graphene-based materials in animal models. Reprinted with permission from ref (24). Copyright 2018 Royal Society of Chemistry.

Since GFNs have a large π-conjugated aromatic structure and high specific surface area, they can be potentially applied in ocular applications, especially for ocular drug delivery systems. High drug loading capacity can be obtained for aromatic containing drugs, such as camptothecin, paclitaxel, and doxorubicin via π–π stacking interactions between graphene layers and drug molecules.^18^ However, in recent years, the potential toxicity of GFNs in biological systems at various levels, including bacteria, fungi, mammalian cells, and animal models as well as their extensive use have been caused a dispute in toxicology research.^19^ The toxicity of GFNs can extremely affect biological systems by their physiochemical properties, including composition, size, shape, surface charge and oxidation status as well as the dose and exposure time. Figure 4 presents the organs in which the main toxic effects (on the right) and biodistribution (on left) of GFNs were found. Until now, there are several studies on the *in vitro* and *in vivo* toxicity of GFNs; however, a comprehensive review on ocular toxicity and applications of GFNs is missing and knowledge about the health risks of eye exposure to the GFNs is predominantly unspecified.^20−24^

For ocular application of GFNs, as mentioned above, it is essential to evaluate their biocompatibility. Since it is inevitable to have an ocular exposure with GFNs for the patients who are utilizing graphene-containing medicines, the workers handling these nanomaterials, and the people who are using graphene-based equipment, it is necessary to enhance the knowledge of the ocular toxicity of GNFs. Consequently, in this review, we looked at the recent advances in the physicochemical properties, ocular toxicity, and ocular applications of GFNs as well as considering the methodologies applied to perform these evaluations.

## 2 Physicochemical Properties of Graphene Family Nanomaterials

The physicochemical properties of GFNs are different from their bulk counterparts and play a major role in their toxicity. Like other organs’ toxicity, the ocular toxicity of GFNs is also strongly affected by their size, lateral dimension, morphology, surface properties, functional groups, concentration, and aggregation states. There are multiple factors that contribute the toxicity of GFNs, but in this section only a few of the principal ones will be discussed.

### 2.1 Size

From a toxicological perspective, one of the prime physicochemical properties that influences GFNs’s toxicity is particle size. Decrease in size to a nano level leads to more cellular uptake due to increasing the surface area and providing further sites for cellular interaction. Several papers reported that the mechanism and the efficiency of cellular uptake, the circulation, distribution, clearance, and toxicity of GFNs depend on the nanoparticles’ size.^25^ The particles with sizes lower than 100 nm can enter the cell, while the particles lower than 40 nm can enter nucleus, and the ones with sizes below 35 nm can cross the blood–brain barrier.^26,27^ For example, compared to GO with a particle size of 780 ± 410 nm, lower cell viability was observed for GO with 160 ± 90 nm at higher concentrations that might be due to increasing reactive oxygen species (ROS) generation in the A549 cell line.^28^ Also, the hemolytic potential evaluation of different sizes of GO and rGO sheets showed that the smaller one had a higher activity, while the minor hemolytic potential was seen for the aggregated rGO.^29^ Morover, the size of GFNs is an important determining factor for subcellular penetration. For instance, rGO nanoplatelets with average lateral dimension of 11 nm can enter the nucleus of human mesenchymal stem cells (hMSCs) and induce more genotoxicity compared to 3 μm nanoplatelets. The micron-sized rGO nanoplatelets showed a high toxicity at high concentration (100 μg mL^–1^) after 1 h exposure time, while the 11 nm rGO nanoplatelets translocated to the nucleus and induced genotoxicity at a very low concentration (0.1–1 μg mL^–1^).^30^

### 2.2 Surface Chemistry

GFNs possess various surface chemistries that lead to different biological activities. The extent of oxidation (O/C ratio) imparts hydrophobic, partially hydrophobic, and intermediate hydrophilic surface chemistry for pG, GO, and rGO, which alter their dispersibility in different solvents and physiologic medium. The hydrophobicity of GFNs causes aggregation because of the π–π stacking between the layers.^31,32^ The cell membranes’ performance and structure can be disrupted by GFNs due to their different surface chemistries. In addition, they are able to stimulate receptors and activate mitochondrial pathways and induce apoptosis.^33−35^

Compared with rGO, GO is enriched by carboxylic acid and hydroxyl and epoxy functionalities at its edges and basal plane. Therefore, owing to the difference in their structures, GO has more dispersion ability, binding sites, and higher activity, which leads to differences in their toxicities. In a study, the ocular toxicity of GO and rGO was evaluated by exposure of GO and rGO to the conjunctival sacs of mice. GO exposure led to observing intraocular inflammation, an incrassated corneal stromal layer, higher corneal stromal cell counts, and TUNEL-positive cells in the cornea as well as iris neovascularization. Unlike GO, by rGO exposure, no specific ocular toxicity was observed, which can be due to their structure and physical characteristic differences.^32^

Additionally, to compare the effect of oxygen level of graphene-based material on its biocompatibility and cytotoxic potential, pG, GO, and low and high oxygen functionalized graphene containing 6.6% and 24% oxygen contents were investigated. ROS generation evaluation was explored using PC12 cells at very low doses of 0.5, 1.0, and 5 μg mL^–1^ for 2 h. The results showed a dose- and oxygen-dependent cytotoxicity response with greater cytotoxicity for pG. The toxicity levels were correlated with an increase in functionalized oxygen content. The ROS measurements showed that the oxidized graphenes, low oxidized graphene (LOG), higher oxidized graphene (HOG), and GO generate higher ROS levels comparing with pG. HOG produced higher superoxide molecules, but free radicals might be reduced due to cellular antioxidant mechanisms of graphene within the cells and its redox ability, preventing the observation of high cytotoxicity.^36^

Another feature that indicates the effect of surface chemistry on cellular uptake is surface charge, the key factor to induce toxicity. The overall charge of cell membrane is negative, which leads to easily binding and ingestion of positive charge nanomaterials to the cell membrane using electrostatic interaction.^37^ Hence, negatively charged GO showed negligible internalization to nonphagocytes;^38^ however, other reports have proposed that the negatively charged nanoparticles internalization into nonphagocytic cells can occur when they bind to the cell surface cationic sites and then they can be taken up by scavenger receptors.^39,40^ Due to the surface charge of nanomaterials, they can absorb several proteins and form “coronas” with proteins in biological systems. Certainly, the affinity and the mode of interaction of the proteins play a determining factor in the formation of protein corona.^41^ Negative charged GFNs cause more electrostatic interaction with the proteins that can alter their circulation, distribution, clearance, and toxicity. Bovine serum albumin-coated GO relieved the cytotoxicity through decreasing its penetration to the cell membrane. Protein corona can decrease GO cellular uptake according to the reported cell viability and cellular uptake results, leading to GO potential cytotoxicity reduction.^42^

The ocular toxicity investigation of PEGylated GO (polyethylene glycol (PEG)-GO) with various oxidation levels and/or surface charges was performed. The results revealed that while the surface charge could change the aggregation status of GFNs, it did not affect the cytotoxicity of PEG-GO samples alone, and the oxidation level had a critical effect on the GO toxicity. Among different PEG-GO samples, GO-h-PEG-NH_2_ (the sample with higher oxidation level) exhibited a higher cytotoxicity compared with the samples with lower oxidation levels.^43^

### 2.3 Surface Modifications

To control the behaviors of GFNs in biological systems and improve their biocompatibility, their surface chemistry plays an important role. Graphene is an extremely hydrophobic compound since it does not have any oxygen-containing hydrophilic functionalities and has the π–π stacking interactions that has crucial implications for its dispersion in water. GO is the most widespread of GFNs because it has been employed for the synthesis of graphene-based nanomaterials on a large scale. GO shows good water dispersion stability and biocompatibility owing to the presence of oxygen-containing functionalities on its edges and surface, which make it a potential nanomaterial for various applications.^44,45^ However, GO still aggregates in physiological buffers because of the charge screening effect of salts.^46^ Consequently, surface modification of graphene and GO is necessary, especially for biomedical applications.^47^ To improve the deficiencies in the structure of graphene and GO, scientists usually introduce active functional groups to improve their dispersion in aqueous and biological media.^10,48−50^

Depending on different application purposes, various surface functionalization strategies have been used to improve GFNs biocompatibility to be used in biomedicine.^51^ Covalent and noncovalent modifications are the most extensive methods for graphene and GO functionalization, aiming to improve their biocompatibility, physiological and colloidal stability, decrease their nonspecific binding to biological molecules and cells, and increase their *in vivo* pharmacokinetics (Table 1).^52−57^ Until now, it is confirmed that GFNs’ functionalization by PEG,^58^ PEGylated poly-l-lysine (PLL),^3^ cyclodextrin,^18^ poly(ε-caprolactone),^44^ poly(vinyl alcohol),^45^ pluronic,^59^ amine,^60^ carboxyl, and dextran^61^ groups significantly decreased their toxicity and improved their biocompatibility.

**Table 1:** Covalent and Noncovalent Modifications of GFNs

modifier	type of modification	goal and application	refs
protein	noncovalent	investigation of cellular effects of GO and identification of the effect of fetal bovine serum on its cytotoxicity	(63)
poly(maleic anhydride-alt-1-octadecene) (C18PMH-PEG5000)	noncovalent	improvement of physiological stability and ultralong blood circulation half-life suitable for photothermal treatment of cancer	(64)
DNA	noncovalent	improvement of water solubility	(65)
gelatin	noncovalent	decrease of the cytotoxicity	(66)
polyethylenimine (PEI)	noncovalent	improvement of physiological stability compared to GO, reduced toxicity compared to pure PEI, and high gene transfection efficiency	(67)
PEGylated phospholipid	noncovalent	improving stability in biological solutions and NIR absorption	(68)
chitosan	covalent	biocompatibility improvement	(29, 69−72)
dextran	covalent	stability improvement of GO in physiological solutions	(61, 73, 74)
PEG	covalent	to improve biocompatibility, reduce nonspecific binding to biological molecules and cells, and improvement of the *in vivo* pharmacokinetics for better tumor targeting	(55, 56, 75−80)
polyacrylic acid	covalent	biocompatibility improvement	(81)
PLL	covalent	biocompatibility and water solubility improvement	(82)
cyclodextrin	covalent	biocompatibility and water solubility improvement	(18)

To investigate the role of GFN functionalities in ocular toxicity, in a study, three kinds of PEGylated GO (GO-PEG-COOH, GO-PEG-OCH_3_, and GO-PEG-NH_2_) were fabricated, and their toxicities on ocular tissue were investigated. Among different PEGylated GO, the GO-PEG-NH_2_ showed the most toxicity to ocular cells. The obtained results of this study can be used for biomedical applications of GFNs in the future by decreasing their toxicity with the aim of suitable surface modifications.^43^ In another study, hydroxylated graphene (G-OH) was prepared, and its ocular biocompatibility was investigated and compare with GO. G-OH displayed some features of GO such as water solubility and processability, whereas it showed higher electroactivity and biocompatibility with human retinal pigment epithelium (RPE) cells than GO.^62^

## 3 Ocular Toxicity of GFNS

Recently, GFNs have attracted much attention in ocular therapeutic delivery and targeting.^3,83^ Since the eyes are very special and sensitive organs, different from most other organs, and the contact between them and GFNs in ocular applications and handling are inevitable, the ocular toxicity of GFNs should be considered. Until now, there are few scientific papers on GFNs’ ocular biocompatibility, and the reports about primary irritation tests for GFNs in the eye are limited, so further research is needed in this field. To date, the cytotoxicity of GFNs on some different parts of the eyes, from the anterior segment to posterior segment of the eyes, has been investigated *in vitro* and *in vivo*. However, different outcomes were reported by these studies due to the differences in experimental models/animals. Consequently, more research studies are needed to investigate the ocular toxicity of GFNs to fill this gap in the research. In the following sections, the toxicity mechanisms of GFNs and the reported ocular toxicity studies of GFNs on different parts of the eyes are presented.

### 3.1 Toxicity Mechanisms of GFNs

**Figure 5:**
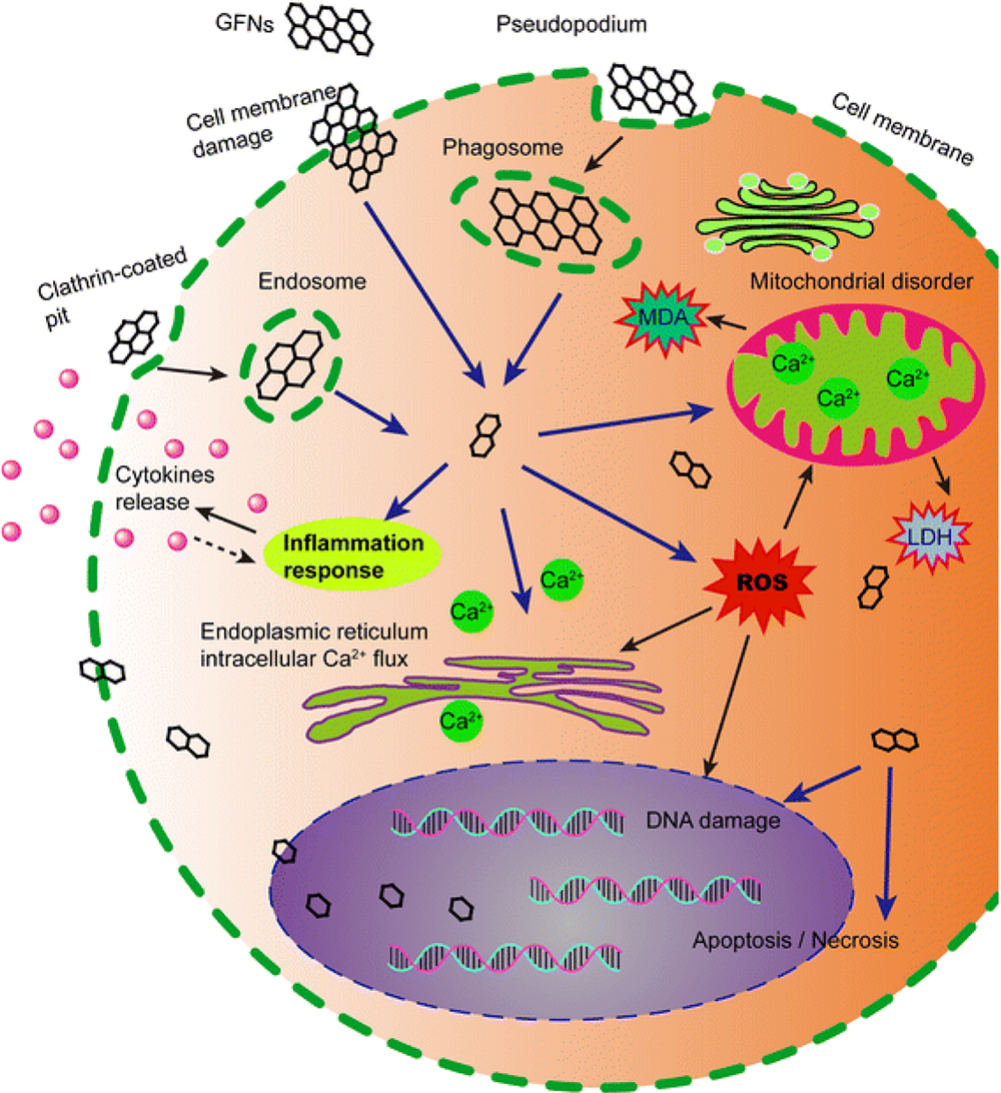
Possible mechanisms of GFNs cytotoxicity. GFNs enter the cells *via* various methods, which induce ROS generation, LDH, increase MDA, and Ca^2+^ release, leading to cell injuries, such as cell membrane damage, inflammation, DNA damage, mitochondrial disorders, apoptosis, or necrosis. Reprinted with permission from ref (27). Copyright 2016 Springer Nature.

The toxicity of GFNs strongly depends on the route of exposure. Depending on their physicochemical properties, including the size, shape, charge, and surface modifications, GFNs have shown different ways to pass through the cell membrane.^84^ As a way of cell entrance, GO can penetrate to the lipid bilayer and enter the cells by adhering and wrapping around them.^85^Figure 5 shows the possible interactions and the main cytotoxicity mechanisms of GFNs.

Although the toxicity mechanism of GFNs has generally been expressed in many studies, the ocular toxicity mechanism of GFNs is complex and still needs to be identified more. The most commonly reported ocular toxicity mechanisms are the oxidative stress, mitochondrial damage, inflammatory response, apoptosis, necrosis, cell membrane damage, cell death, cell cycle disorder, and cell viability loss.^32,86−90^

GFNs are able to bind with the surface of the cell membrane due to their favorable surface curvature, leading to abnormal stretching of the cell membrane and cytotoxicity.^91−93^ Moreover, by overwhelming the antioxidant enzymes, GFNs can lead to excessive ROS generation levels.^94−96^ In addition, GFNs can lead to a significant inflammatory response through release of cytokines and chemokines, which cause the recruitment of circulating monocytes and stimulate the secretion of Th1/Th2 cytokines and chemokines.^97,98^ The formation and cytotoxicity of ROS is not limited to GFNs, but in many GFNs, it is the first mechanisms that causes toxicity.^99,100^ Superoxide dismutase or glutathione peroxidase enzymes as antioxidant enzymes can decrease and remove ROS. Generally, ROS can be generated due to different reactions including GO and rGO nanosheets reaction with H_2_O_2_ to form hydroxyl radicals, which is known as a Fenton reaction and charge transfer between GFNs and other redox-active agents.^8,101−106^ GFNs have shown cell membrane damage owing to their physical properties, including size and hydrophobic surface properties. They can significantly cause cytotoxicity by interacting with cell membrane lipids, leading to the morphological extension of F-actin filopodial and cytoskeletal dysfunction. In addition, GFNs sharp edges, known as “blade”, act, insert, and cut through cells’ membrane.^107−110^ Cell exposure to GFNs can also lead to a considerable increase in mitochondrial oxygen consumption and elimination of the potential mitochondrial membrane that ultimately causes apoptosis by activating the mitochondrial pathway.^111−116^ Moreover, GFNs can cause tissue injury and an inflammatory response with the secretion of cytokines and chemokines. Also, inflammatory responses or cellular injury can lead to apoptosis/necrosis.^117−119^

### 3.2 Toxicity of GFNS on the Anterior Segment of the Eye

Until now, the toxicity of GFNs on some anterior segment of the eyes, including conjunctiva, cornea, iris, and lens, has been investigated. In patients with end-stage corneal blindness, synthetic keratoprostheses are required for visual rehabilitation. To this aim, Tan et al.^86^ used two types of graphene, graphene film (G-film) and graphene foam (G-foam), for the synthetic keratoprosthesis skirt and assessed their biocompatibilities by *in vitro* cell culture using human corneal stromal cells and an *in vivo* rabbit implantation model. For *in vitro* assessment, human corneal stromal fibroblasts were cultured on the surface of G-film, G-foam and pristine titanium (Ti) discs as a standard, and a tissue culture plastic surface (TCPS). Good biocompatibility with human stromal fibroblasts was observed for G-film in terms of cell adhesion, viability, and proliferation. The number of cells was higher on G-films compared with TCPS control, and 10% more cell proliferation was seen on graphene in comparison with on Ti. Moreover, compared with Ti and G-film, the culture medium collected from fibroblasts seeded on G-foam demonstrated lower cytokines (IL-6 and IL-8), which can be due to a lower expression of cytokines by cells, or adsorption of the inflammatory signal molecules with graphene materials. No sign of infection, neovascularization, or inflammation was seen by the implantation of G-film into rabbit stroma, confirming short-term biocompatibility of graphene with corneal cells and tissue, which can be developed for cornea tissue engineering.

**Figure 6:**
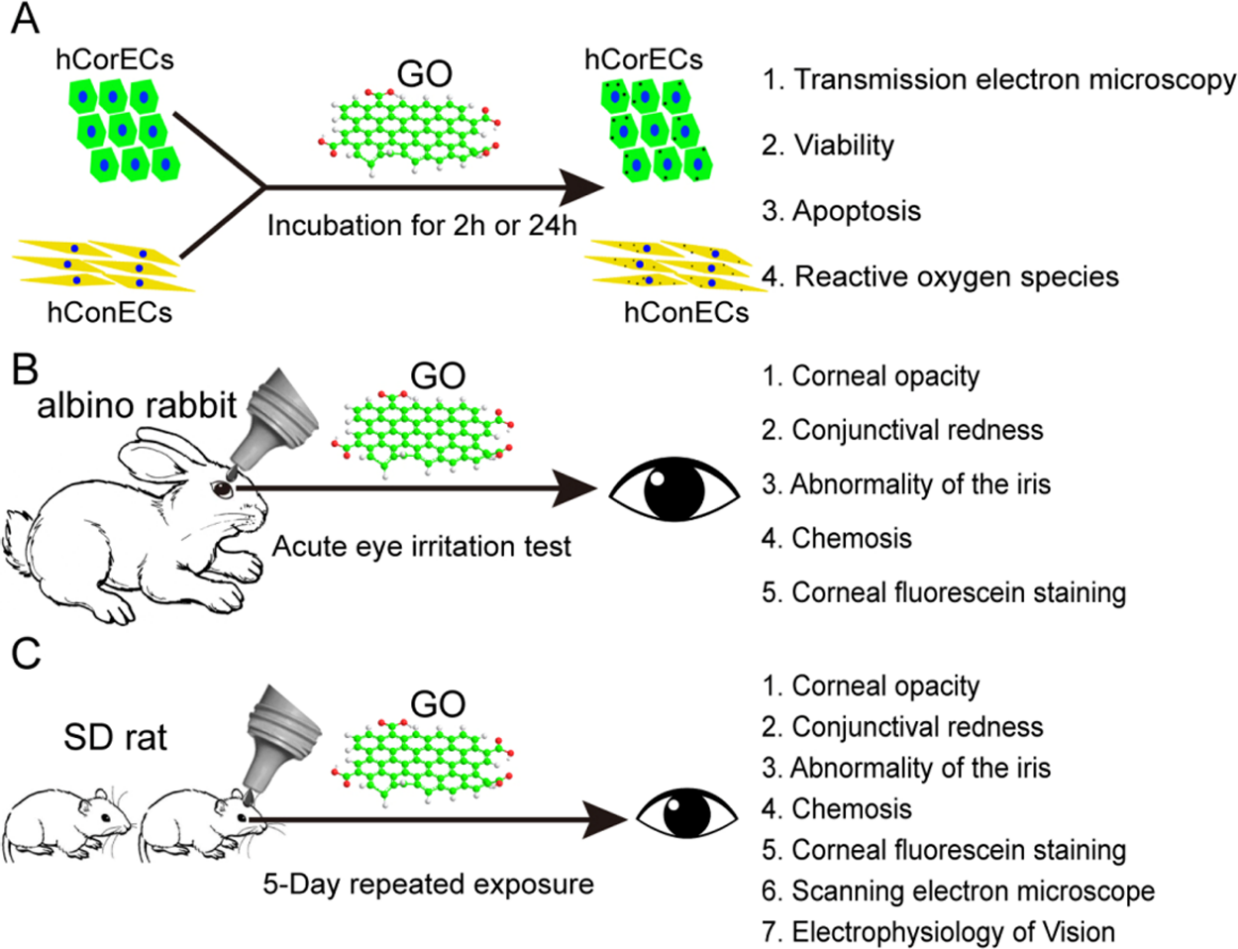
*In vitro* (A) and *in vivo* (B, C) experimental illustrations to evaluate the potential of GO ocular irritation. Reprinted with permission from ref (87). Copyright 2016 Taylor & Francis.

Organisation for Economic Co-operation and Development (OECD) guidelines for the Testing of Chemicals (Test No. 405) can be used as a standard for investigation of acute eye irritation/corrosion. According to these guidelines, eye irritation is defined as “... the production of changes in the eye following application of a test substance to the anterior surface of the eye, which are fully reversible within 21 days of application”. Moreover, the eye corrosion is also defined as “... the production of tissue damage in the eye, or serious physical decay of vision, following application of a test substance to the anterior surface of the eye, which is not fully reversible within 21 days of application”. Aiming to this guideline, the time- and dose-dependent cytotoxicity of GO through oxidative stress was reported by Wu et al. using human corneal epithelium cells (hCorECs) and human conjunctiva epithelium cells (hConECs).^87^ The acute eye irritation tests were performed in this study in albino rabbits based on the OECD guidelines. They investigated the influence of GO exposure to the ocular surface *in vitro* and *in vivo*, considering different concentrations (12.5–100 μg mL^–1^) and times of exposure (Figure 12). Although, no cytotoxicity to hCorECs was seen by acute GO exposure (2 h); however, significant cytotoxicity to hCorECs and hConECs was observed through short-term GO exposure (24 h) with higher ROS generation (Figure 6A). The obtained results revealed that no sign of corneal opacity, conjunctival redness, abnormality of the iris, or chemosis was seen in the rabbits after the instillation of 100 μg mL^–1^ of GO (Figure 6B). However, reversible mild corneal opacity, conjunctival redness, and corneal epithelium damage were shown for 5-day repeated GO exposure (50 and 100 μg mL^–1^) in Sprague–Dawley rats that was alleviated by glutathione (GSH) (Figure 6C). Therefore, no acute eye irritation was seen through occasional GO exposure; however, short-term repeated GO exposure led to reversible damage to the eye through oxidative stress that can be reduced by the antioxidant GSH.

In another study on GFNs, the right conjunctival sacs of the Kunming mice (female) were exposed once per day for a total of 7 days to the rGO and GO suspensions.^120^*In vivo* and *in vitro* morphological and molecular biological analysis revealed no significant ocular toxicity for rGO, while different signs of toxicity, such as intraocular inflammation, an incrassated corneal stromal layer, cell apoptosis in the cornea, iris neovascularization, and significant cytotoxicity of rat corneal epithelial cells were seen by short-term GO exposure. After short-term GO contact with eyes, many lymphoid cells were produced, as shown by the HE staining results. Interleukin expression was induced by lymphoid cell proliferation. In addition, compared with the control group, in the GO treatment group, the level of IL-6 and IL-8 expressions were considerably higher. In addition, the TNF-α expression level, which is one of the important inflammatory factors, was also evaluated *in vivo* using GO and rGO. In the GO model mice, the TNF-α content was considerably enhanced in the eye, while it did not change in the rGO model mice. Therefore, the inflammatory response had been triggered by increasing the IL-6, IL-8, and TNF-α in the GO model mice. Moreover, the oxygenation levels of eyes in the three groups of mice were indicated by the malondialdehyde (MDA) content. In addition, the *in vitro* results demonstrated that the main patterns of GO cytotoxicity during cell injury were necrocytosis, cell death, cell cycle disorder, and cell viability loss.

The toxicity investigation of PEG-GO with various oxidation levels and/or surface charges including positive, negative, and neutral charge on hCorECs and intraocular cells (hRCECs) was performed. The results revealed that the viability of both cell types decreased by increasing PEG-GO concentration. Among different PEG-GO samples, the GO-h-PEG-NH_2_ sample, which had a higher oxidation level, exhibited higher toxicity. To understand the potential toxicity mechanisms and complicated interactions between GFNs and biological systems on a whole cell level, a study of gene expression profiles can be used as an important approach. Therefore, in this study, the gene expression profile was studied and the results demonstrated that the accumulation of ROS induced by GO-h-PEG-NH_2_ treatment was attributed to NDUFB9-mediated biological pathway.^43^

### 3.3 Toxicity of GFNs on Posterior Segment of the Eye

**Figure 7:**
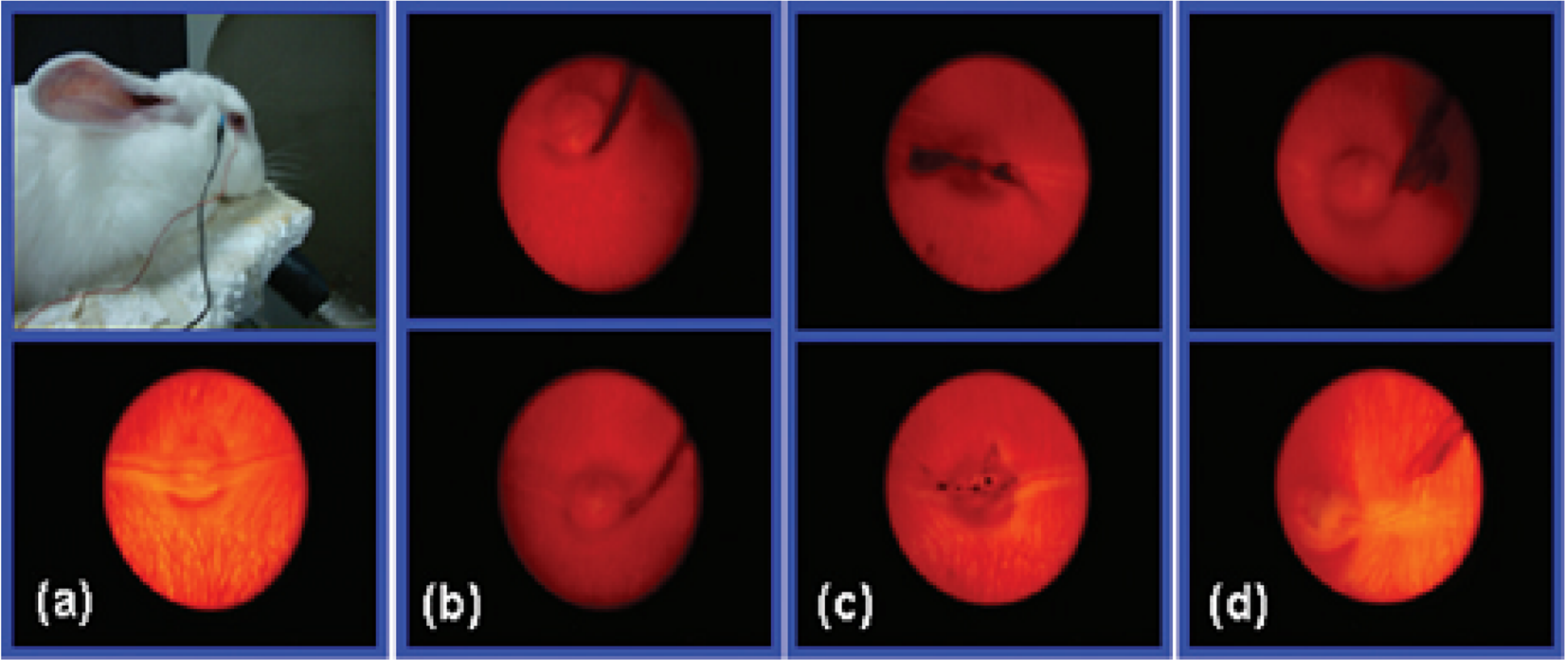
(a) Digital photos of the experimental rabbit (top) and slim-lamp fundus photo of the control eye (bottom). Slim-lamp fundus photos of the eyes after 2 (top) and 49 days (bottom) intravitreally injection of (b) 0.1, (c) 0.2 and (d) 0.3 mg of GO. Reprinted with permission from ref (88). Copyright 2012 American Chemical Society.

The toxicity of GFNs on some posterior segment of the eye was also investigated, such as retina, macula, and optic nerve. The effect of GO on RPE cells in terms of the cell morphology, viability, membrane integrity, and apoptosis was examined by Yan et al., using several techniques, such as optical micrography, cell counting kit-8, lactate dehydrogenase (LDH), and apoptosis assays.^88^ RPE cells exhibited >60% cell viability in GO solutions and <8% LDH release. Although LDH release into the culture medium upon cells showed a very low impact on cell morphology, after a long-time culturing, the change was noticeable as well as aggregation of GO. Moreover, the results showed a negligible cell apoptosis (∼1.5%) with the addition of 100 μg mL^–1^ GO (similar to the control cells), indicating GO biocompatibility. The biocompatibility of GO was also investigated *in vivo* by intravitreally injection of 0.1, 0.2, and 0.3 mg of GO into white rabbits’ eyes. One eye was injected with GO, and the salt solution (balance salt solution) was injected to the other eye of the same rabbit as the control. As seen in Figure 7, in all experimental groups, after 2 and even 49 days, no ocular changes were visible. Compared to the control eye (Figure 7a), corneas, anterior media, posterior media, and retinas of the rabbits’ eyes were clear without signs of inflammation even after GO injection for 49 days (Figure 7b–d, bottom). Furthermore, since the time increased from 2 (Figure 7b–d, top) to 49 (Figure 7b–d, bottom) days, the amount of GO was reduced gently in the eyeballs, which can be due to GO diffusion in the vitreous. According to the reported results, the intravitreal injection of GO also did not cause any changes in intraocular pressure (IOP), eyesight, and electroretinogram measurements.

In addition, the histopathology studies of the rabbits’ eyes revealed that while a very low content of GO remained in the GO-injected eye, by comparing with the control eye, no retinal abnormality was seen. These observations suggested that by GO injection, no apparent damage occurred to the retinal morphology. Considering both the *in vitro* and the *in vivo* results obtained in this study, it can be concluded that there is no severe GO toxicity on the eyes.^88^ However, still more studies, such as controlling GO aggregation by surface functionalization and GO genotoxicity as well as the impact of physicochemical properties on the GO toxicity, should be considered before making a certain conclusion about the safety of GO.

In another research work, GO showed mitochondria damage and induced developmental malformation of the zebrafish eyes.^89^ Hydroxylated graphene (G-OH) showed genotoxicity at 4100 mg mL^–1^ concentration. Although the genotoxicity of G-OH in rabbits was decreased gradually through intravitreal injection after 4 weeks, it did not show damage to cell morphology, structure, and most parts of the eyes. It led to IOP, ERG, and retinal structure changes as eyesight-related functions. The cytotoxicity mechanism results also revealed that through endocytosis and exocytosis, G-OH could penetrate into and out of the cytoplasm without any cell membrane damage.^90^

GFNs can be used in ophthalmology to treat different ocular disorders. Recently, Zambrano-Andazol et al.^121^ developed rGO membrane (rGOM) for ocular regenerative medicine application. For ocular tissue engineering, the used membrane should have cellular biocompatibility and promote wound healing as well as show antimicrobial properties. Because of these needed criteria, the *in vitro* and *in vivo* biocompatibility and genotoxicity of rGOM were investigated using various human ocular cells. The results demonstrated that no sign of cytotoxicity or genotoxicity was seen after short-term exposure of rGOM compared with control group cultures and allowed the growth of different ocular cells. Although the obtained results of this study were very promising, a long-term follow-up period and additional *in vivo* research are needed to find out whether rGOM can be a good candidate for treatment of ocular diseases or not.

## 4 Ocular Applications of GFNS

### 4.1 Ocular Therapeutic Applications

Recently, graphene and GO exhibited compatible physicochemical properties, which makes them suitable for biomedical applications, such as drug and gene delivery, biosensing and imaging, and tissue engineering.^122,123^ Ocular drug delivery using GFN-containing systems has attracted a continual interest of researchers in the past few decade. Nanocomposite oleogels of groundnut oil and stearic acid containing different percentages of GO were developed to improve corneal permeation of ciprofloxacin HCl (CPH), an antibacterial drug, by Hasda et al.^124^ The *in vitro* release study showed that by increasing the GO content up to 0.05% in the nanocomposite oleogels, a higher cumulative percentage of drug permeation through caprine cornea was seen. Moreover, by incorporation of GO in the oleogels, a higher *ex vivo* corneal permeation of CPH by Fickian diffusion model was obtained.

To treat and manage various corneal diseases such as keratoconus and dry eye syndrome, eye drop solution is widely used. However, due to a frequent dosing schedule, it can affect the routine lifestyle of patients. In one study, hyaluronic acid (HA) and rGO loaded silicon contact lens were developed for corneal epithelial healing, which has the potential for improving the tear fluid volume for controlling numerous ocular diseases, such as dry eye syndrome.^125^ Silicon contact lenses containing HA and rGO were prepared through direct loading of HA and rGO in the contact lens (HA-GO-DL) and also by the conventional soaking method (HA-GO-SM). The contact lenses containing lower amounts rGO exhibited acceptable swelling and transmittance properties. In addition, the HA-GO-DL contact lenses demonstrated a water retention property according to the water evaporation study results. Moreover, the flux data revealed that the HA-GO-DL lenses showed low burst with sustained release up to 96 h, whereas a high burst release was seen for HA-GO-SM lenses after 24 h. Furthermore, the results of ocular irritation study confirmed the safety of the HA-GO-DL lenses. In comparison to HA-GO-SM and eye drop solution, a high HA-tear fluid concentration was seen by using the HA-GO-DL batch as well as improvement in the fluid volume of rabbit tears.

**Figure 8:**
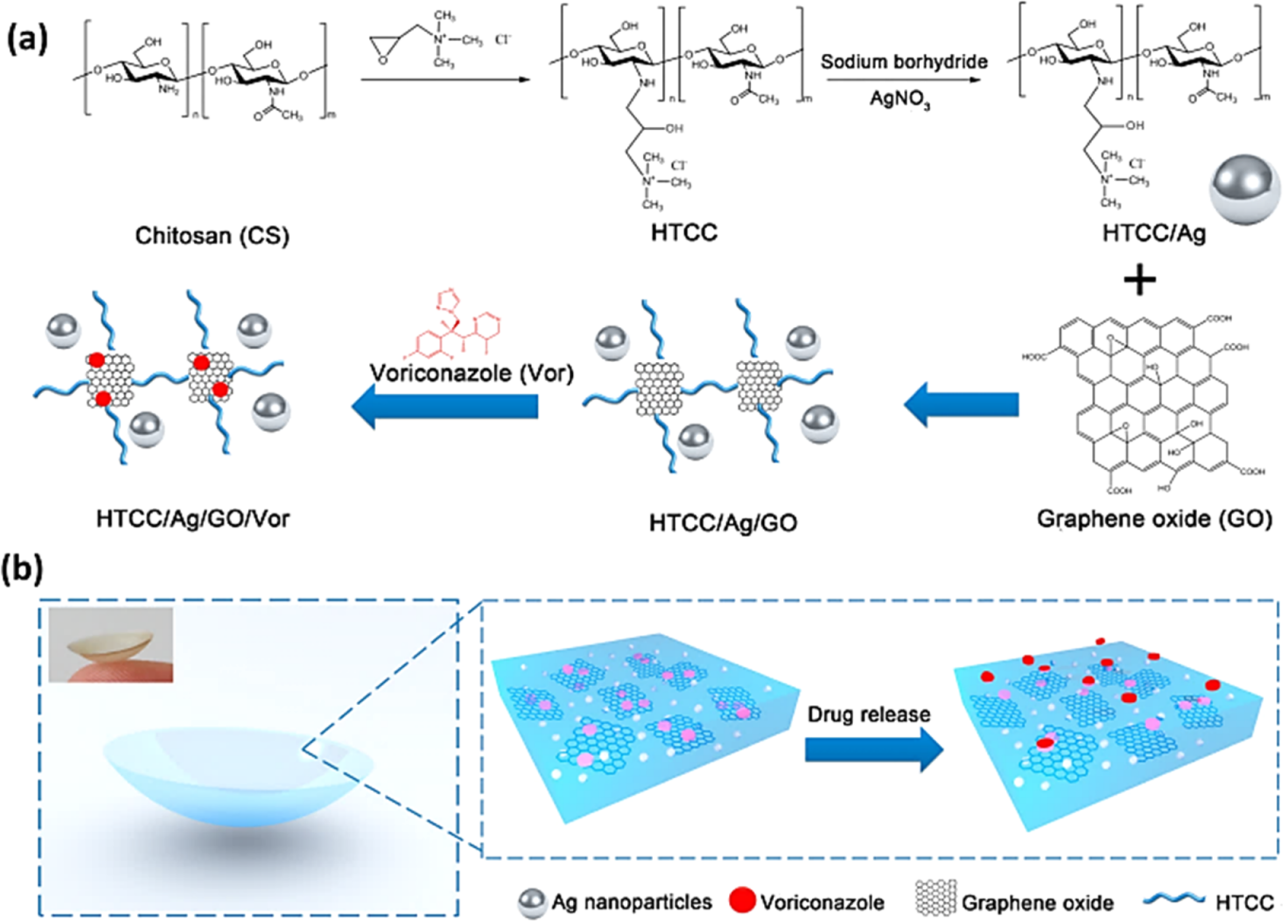
(a) HTCC/Ag/GO/Vor synthesis pathway. (b) Schematic demonstration of drug loaded contact lenses and controlled drug release. Reprinted with permission from ref (127). Copyright 2016 American Chemical Society.

Recently, an active targeted drug delivery via endocytosis mediated by ligand–receptors was used for doxorubicin (DOX) delivery to choroidal melanoma. Transferrin (Tf)-modified pegylated graphene nanocarrier was used for DOX loading and provided a targeted capability toward ocular choroidal melanoma-1 cells, which typically exhibited a high expression of Tf receptors. DOX was loaded into the nanocarrier through π–π stacking architecture. The results displayed more than 80% tumor cell inhibition, which confirmed the potential of using Tf-PG-DOX as an extremely effective antitumor drug delivery system suitable for choroidal melanoma.^126^ Owing to the very limited therapeutic efficacy of customary treatments, such as eye drops and probable intraocular injection side effects, hydrogel contact lenses have been considered as suitable ocular drug delivery systems due to their comfortable structure and drug loading capacity. Considering this, in one research study, quaternized chitosan (HTCC), silver nanoparticles, and GO were used for development of hydrogel-based contact lens, which had both antibacterial and antifungal activities.^127^Figure 8 shows the synthesis pathway of HTCC/Ag/GO as a voriconazole (Vor) delivery system with integrated antifungal functions. The GO physical structure and hydrophobic property can lead to an increase in drug loading capacity and prolong drug release time. The antimicrobial activity and cell viability assessment of Ag loaded contact lens containing GO demonstrated good activity with no toxicity, which were comparable to those of the untreated cells.

**Figure 9:**
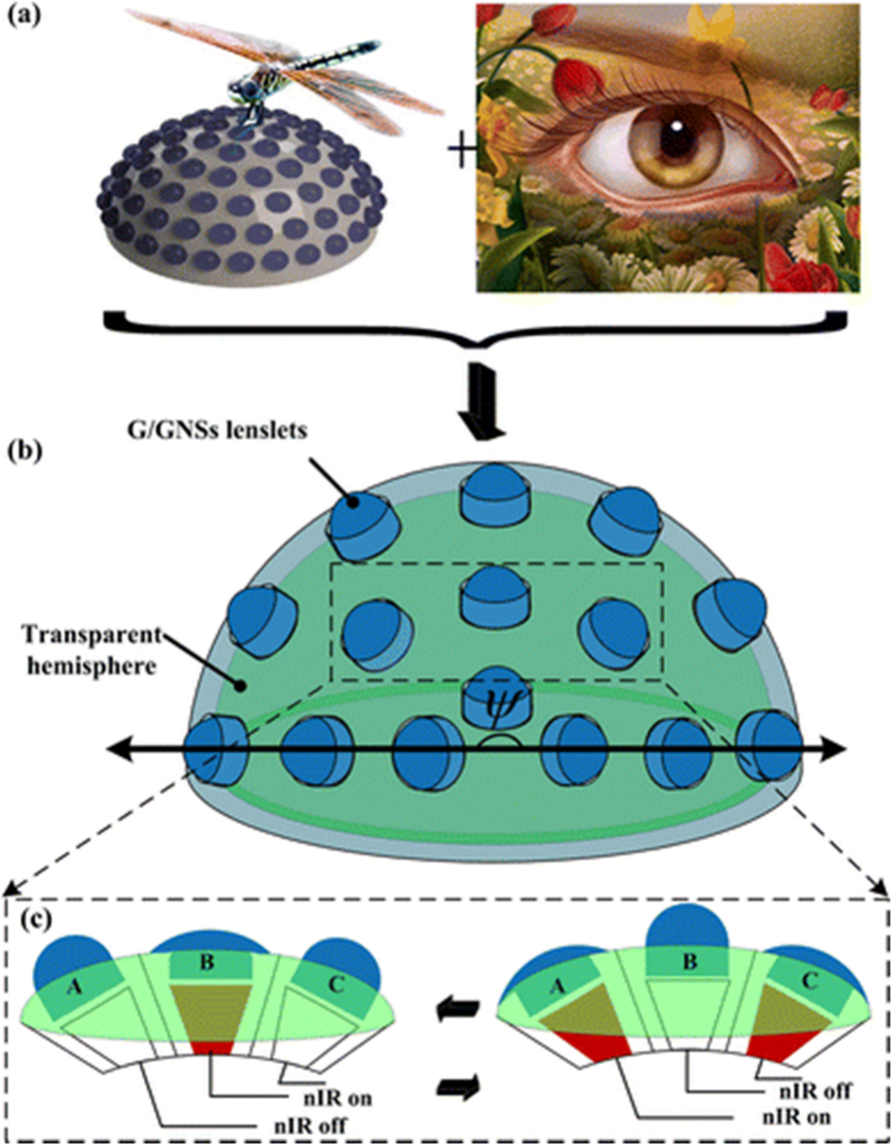
Schematic illustration of wide FOV and vari-focal ability of BCEs: (a) Panoramic FOV and varifocal ability come from compound eyes of insects and single-lens eyes of vertebrates, respectively. (b) To realize the panoramic FOV (ψ), high-density lenslets were omnidirectionally distributed on a hemispheric dome. (c) The performance of GNSs as a ciliary muscle provided programmable vari-focusing and remote actuating for BCEs under nIR irradiation. Reprinted with permission from ref (128). Copyright 2015 American Chemical Society.

Bioinspired compound eyes (BCEs) as micro-optical devices are tremendously interesting due to their large fields of view (FOV) and various focal capability. Wang et al. prepared a glycerol/graphene nanosheets (G/GNSs)-based BCEs through a template-directed self-assembly process (Figure 9). For light-actuated BCEs, GNSs were used with large FOV and ability of programmable focusing. G/GNSs lenslets were homogeneously arranged on a hemispherical dome that provided a large FOV up to 160°. GNSs enabled BCEs to show a reversible 4-fold zoom and programmable varifocal under remote near-infrared (nIR) laser light irradiation (Figure 9c) led to development of tunable lenslet similar to human eyes. The photothermal conversion of GNSs causes nIR pulsed laser absorbing and a change to thermal energy, enhancing the lenslets’ temperature and adjustment of lenslet curvature.^128^

One-step laser reduced GO conductive tracks on transparent and flexible poly(ethylene terephthalate) substrates also showed increased absorption toward a shorter wavelength of up to 96% in UV regions, which can significantly protect human eyes from high-energy light hazards.^129^

### 4.2 Ocular Diagnosis Applications

Currently, contact lenses are used for cosmetic reasons and correction of vision. A unique platform can be provided for ocular diagnostics through continuous contact of contact lenses with our tear fluids.^130^ Hence, researchers have developed contact lenses using electronic devices for detecting physiological changes for the diagnosis of diseases.^131^ To develop electronics on soft contact lenses, optical transparency, stretchability, flexibility, and reliability upon repeated eye-blinks for clear vision are the important demanding challenges. To overcome these challenges, they can be worn and produced by transparent, stretchable materials and harmless to the human body. Regarding the development of wearable soft contact lenses, graphene and its hybrid with metal nanowires were used as multifunctional sensors with suitable transparency and stretchability for wireless diagnosis and management of diabetes and IOP. Indeed, graphene and its hybrid provided enough transparency and stretchability, which makes sure for the users that the soft contact lens is reliable and comfortable for unobstructed vision when it is worn.^132^ Recently, flexibility and transparency of graphene led to development of liquid contact lenses with a large FOV, a compact size, and fast response to electric potential.^133^

Ocular hypertension is the most important risk factor in glaucoma, which has a higher IOP than normal.^134^ High IOP causes loss of peripheral visual fields and leads to irreversible loss of vision fields.^135^ Unfortunately, glaucoma caused by high IOP is the second main cause of blindness in the world. As a result, measuring or monitoring the IOP is important for glaucoma inhibition and management. Up to now, many tonometers, the main tool to measure IOP, have also been developed; however, they can be used by professionals for repetitive measurements to identify spikes and fluctuations of the patients’ daily IOP.^136,137^ Zeng et al. developed an IOP sensor using transparent graphene to prevent the sensors from blocking vision and enhancing the flexibility of the wearable sensors to fit several sizes or curvatures in the eye.^138^ Electrooculography (EOG) is a technique to record the cornea-retinal standing potential induced by eyeball movements. EOG applications are an ophthalmological diagnosis and can be used for developing wearable medical sensors and as human–computer interaction interfaces.^139,140^ To resolve the limitation of silver/silver chloride (Ag/AgCl) gel-based, “wet” electrodes, a common method to measure the biopotentials, the graphene-coated textile electrodes were developed for monitoring cardiac biopotentials. Graphene textile electrodes with a high degree of flexibility and stretchability can be used in various kinds of personal clothing to monitor the epileptic patients and driver drowsiness, diagnostic polysomnogram tests for sleep disorders, and for developing wearable human–computer interfaces.^141^ Regarding the importance of visual electrophysiology measurements, soft graphene contact lens electrodes were used for conformal, full cornea recording of electroretinography (ERG) from cynomolgus monkeys. ERG is a test to measure the electrical potential changes at the corneal surface, which is employed in ophthalmic diagnostic testing for the assessment of retina functional integrity. The softness and optical transparency of graphene increased the high-efficacy measurements of various kinds of ERG signals, including full-field ERG, multifocal ERGs, and multielectrode ERG, with negligible corneal irritation.^142^ Besides, electrical signals in mouse retina were investigated using a combination of field-effect transistors containing graphene and scanning photocurrent microscopy with microfluidic platforms to detect the neural activity of retina. Results showed that graphene concentration in the carrier can be modulated by electrical activity in living retinal tissues, leading to potential gradients that can separate photoexcited electron–hole pairs and produce photocurrent signals.^143^

**Figure 10:**
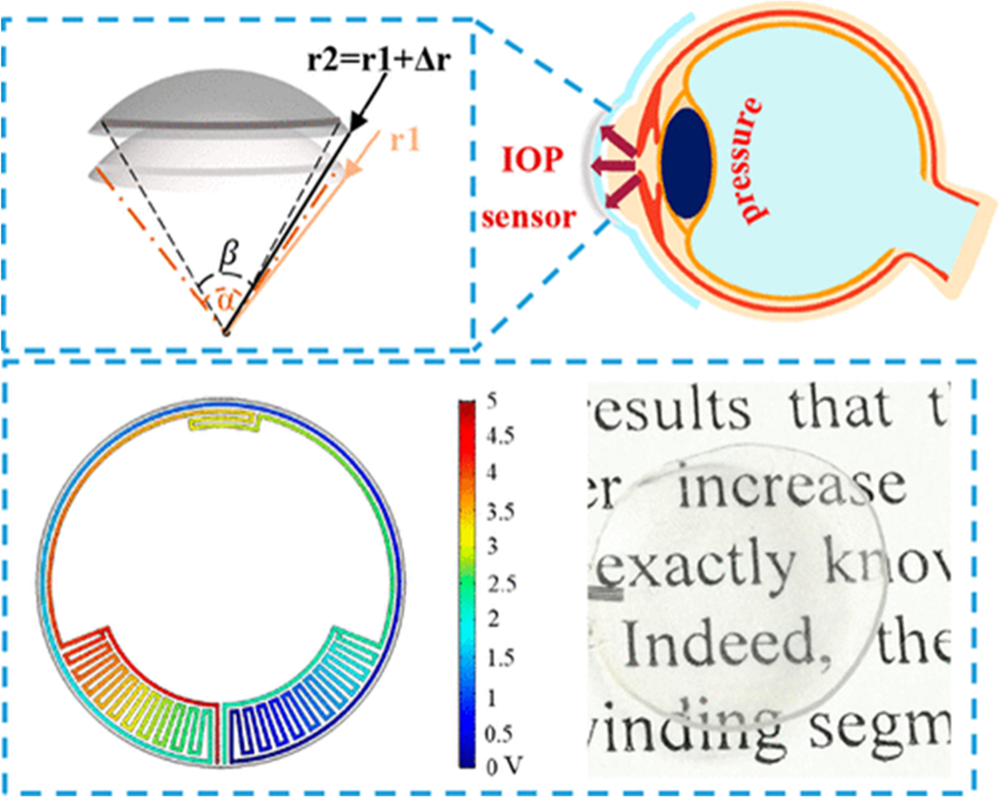
Design and simulation of the IOP sensor. Reprinted with permission from ref (144). Copyright 2020 American Chemical Society.

A highly transparent, sensitive, and wireless sensor using graphene was developed for continuous and noninvasive IOP monitoring by Xu et al.^144^ In this study, FLG was used for development of a sensor with high transparency, sensitivity, linearity, stability, durability, reliability, and biocompatibility for 24 h monitoring of IOP (Figure 10). The IOP sensor operation on a silicone eyeball was tested, confirming the relevance of its output voltage with the IOP fluctuation. Furthermore, the designed wireless sensor system can be used to monitor the IOP using a mobile phone. Consequently, the prepared sensor can be used for glaucoma diagnosis and treatment owing to its average transparency of 85%, simple preparation method, and its capability for continuous monitoring of IOP.

In addition, recently, a wearable contact lens sensor for noninvasive in situ monitoring of IOP was developed by Fan et al.,^145^ using flexible polydimethyl siloxane (PDMS) and parylene-containing rGO and CNT. The sensing performance of prepared contact lens sensor showed a high sensitivity of 36.01 μV mmHg^–1^ using an eyeball model made of PDMS to simulate the curved surface of human eye. High sensitivity to IOP change, good linearity, good accuracy and great stability within the clinically relevant IOP range were the outstanding properties of the fabricated sensor. In another study, a contact lens for detection of IOP was developed using three-dimensional graphene nanowalls (GNWs) through the gold-assisted transfer method. The resistance response of the developed sensor to the normal IOP fluctuation was 1.014 kΩ mmHg^–1^ with a normal sensitivity of 42,250 ppm mmHg^–1^ and the response range of 0–75 mmHg according to the simulated tests on porcine eyes *in vitro*. The obtained results revealed that the GNWs have significant potential for continuous IOP monitoring with high sensitivity and low power consumption.^146^

**Figure 11:**
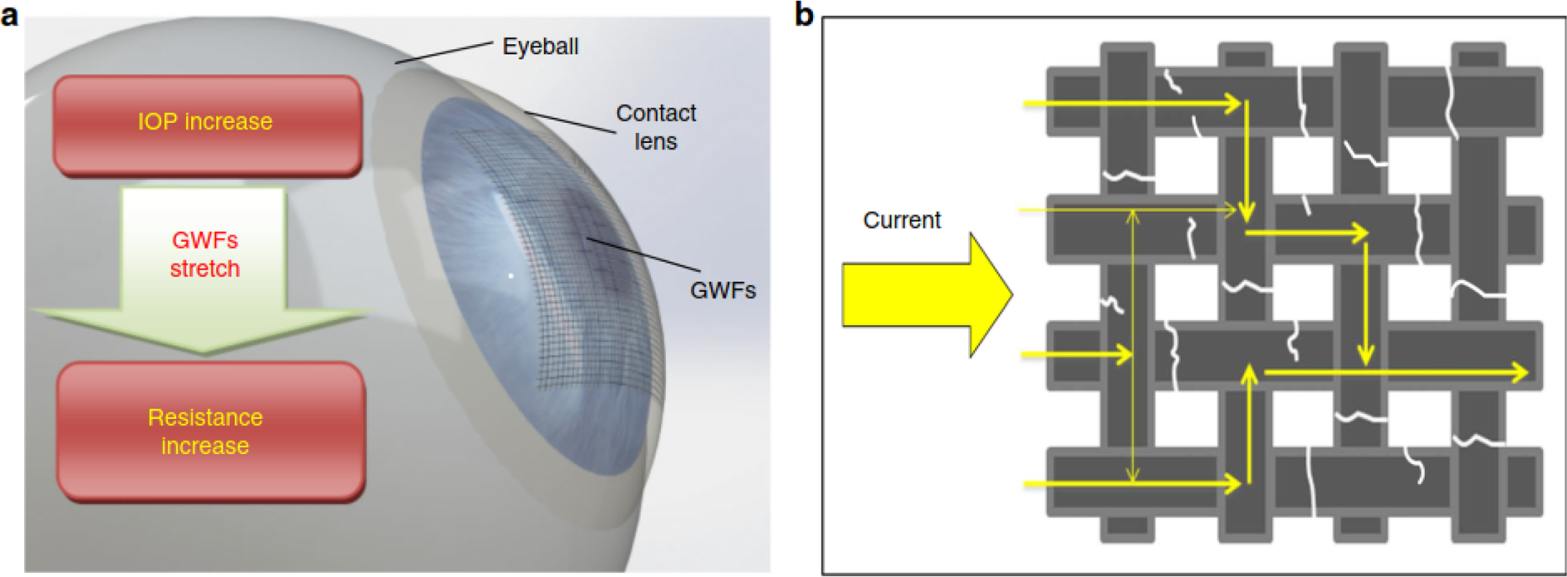
(a) The principle performance of the contact lens containing GWF. (b) Current pathway through a fractured GWF. Reprinted with permission from ref (147). Copyright 2019 Springer Nature.

Graphene woven fabrics (GWFs) were also employed in contact lenses by Zhang et al.^147^ to monitor the IOP (Figure 11). The GWF-containing contact lenses demonstrated excellent sensitivity of resistance to strain, flexibility, stretchability, transparency, and biocompatibility, which can be used for real-time IOP monitoring with high resolution. The *in vitro* results revealed the effectiveness of the prepared device by evaluating the changes in resistance rate under various IOP. However, *in vivo* tests and studies of the long-term reliability and stability still need to be done for further confirmation of the device’s capability for using in clinics.

**Figure 12:**
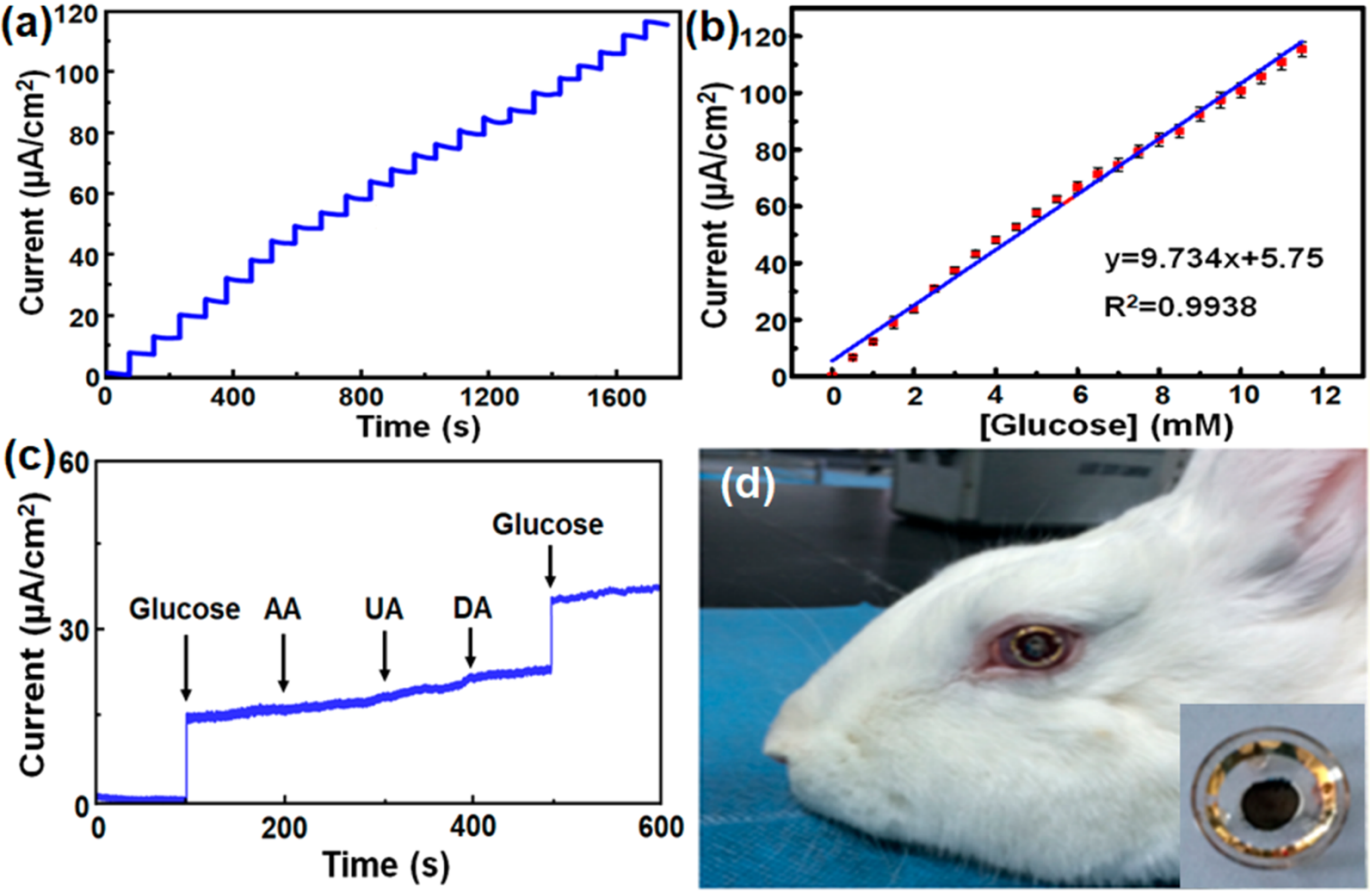
Evaluating the biosensing function of the biosensor containing GC-COOH. (a) Amperometric response of the GC-GOx enzyme electrode to the addition of 0.5 mM glucose at +0.5 V vs Ag/AgCl in 0.1 M PBS (pH = 7.4). (b) Calibration curve for detection of glucose. (c) Amperometric response of the GC-GOx enzyme electrode to the addition of 1 mM glucose, 50 mM uric acid, 50 mM ascorbic acid, and 50 mM dopamine at +0.5 V vs Ag/AgCl in 0.1 M PBS (pH = 7.4). (d) GC-COOH-based intraocular biosensor worn on a rabbit’s cornea. The inset in (d) displays the biosensor working side. Reprinted with permission from ref (148). Copyright 2020 American Chemical Society.

In addition to contact lenses, recently, a high-performance intraocular biosensor made of carboxylated chitosan-functionalized nitrogen-containing graphene (GC-COOH) was fabricated for detection of glucose sugar from tears.^148^ In general, it is crucially important for diabetic patients to detect their glucose sugar level for early treatment, so the noninvasive and real-time detection from tears will be very valuable. In this study, a high-performance intraocular biosensor containing nitrogen-doped graphene was developed, which showed a high electroactive property that can act as an ophthalmic electrode. The fabricated chitosan-graphene based biosensor showed a high sensitivity at 9.7 μA mM^–1^ cm^–2^, a broad linear range at 12 mM, and a good detection limit of 9.5 μM. The synthesized graphene-based biosensor also remained stable after a month of storage. The *in vitro* ocular biocompatibility of GC-COOH was investigated using CECs and RPE cells. The as-prepared GC-COOH was highly biocompatible to ophthalmologic cells. Moreover, the effect of biosensing electrode was examined on ocular tissues *in vivo*, and to monitor the intraocular blood sugar in tears, the electrode was evaluated as an assembled wearable corneal contact electrode using New Zealand white rabbits as animal models (Figure 12).^148^ According to the obtained results, there were no changes in the IOP or the corneal structure. The developed sensor was worn by the animals for more than 24 h without any inappropriate influence. The obtained results of this study approved the potential of the biosensor for clinical intraocular applications.

## 5 Concluding Perspectives

Over the past few decades, GFNs have been explored for ocular therapeutic and diagnosis applications. The high specific surface area and large π-conjugated aromatic structure of GFNs make them good candidates for the development of ocular materials and devices, such as ocular drug delivery systems and sensors. Besides their advantages, there are some challenges regarding GFNs’ toxicity especially for clinical use. Currently, since the literature about ocular toxicity of GFNs is limited, it is hard to conclude the potential GFNs’ ocular hazards. Until now, there are two opposite opinions in this research field: Some researchers suggested that GFNs are biocompatible and can be good candidates for ocular applications,^86,88,121,124^ while others reported unfavorable biological responses and cytotoxicity.^32,90^ These inconsistent results might have been caused by differences in research groups, experimental models/animals, and physicochemical characterizations of GFNs and their compositions. Once GFNs are prepared and selected for ocular applications, their biocompatibility should be evaluated, and further detailed and accurate experiments regarding toxicity of GFNs must be done.

In most of the studies detailed herein, the toxicity of GFNs extremely depends on their physicochemical properties, including size, surface functional groups, oxidative state, and dose of administration as well as exposure time. GFNs are very large family with huge differences in size and dimension, which affect the toxicity. In addition, studies frequently showed that unmodified graphene and GO were more cytotoxic compared with functionalized GFNs and rGO. Therefore, useful surface modification must be carefully evaluated and used to decrease the GFNs’ cytotoxicity for ocular applications in the future. Surface functionalization of GFNs using biopolymers, such as PEG, can led to an increase their biocompatibility in ocular applications and improve their therapeutic effects. Another physicochemical property that can influence the ocular toxicity of GFNs is degree of oxidation. A promising approach for reducing the GFNs’ ocular toxicity and improving their safety for ocular applications is minimizing the degree of oxidation.

In addition, another important issue that needs to be considered is the long-term fate of GFNs after entering the eye or being taken up by cells. In most of the reported studies, the short-term ocular toxicity has been assessed, but long-term follow-up period should be considered. Therefore, extended research is needed to evaluate whether longer treatment times can affect the toxicity of GFNs in ocular applications or not.

To improve the safety of using GFNs in ocular applications, it is essential to study their toxicity mechanisms. To date, various ocular toxicity mechanisms of GFNs have been investigated and extensively approved, including mitochondrial damage, oxidative stress, inflammatory response, apoptosis, necrosis, cell membrane damage, cell death, cell cycle disorder, and cell viability loss. However, more specific pathways of the ocular toxicity mechanism of GFNs need to be discovered and investigated. For a better understanding of complex interactions between GFNs and biological systems, gene expression profiles should be studied as an important approach, which reveals the potential molecular mechanisms of toxicity on a whole cell level.

Moreover, hydrogels containing GFNs have been extensively employed and studied in contact lenses since they are highly comfortable and biocompatible and have a high surface area. However, there will be some drawbacks by changing some conditions, such as increasing the content of GFNs and time of exposure. In addition, selecting the right cell lines and/or animal model in assessment of ocular toxicity is crucially important to develop ocular formulations with proper safety and efficiency.

In conclusion, since ocular devices based on GFNs are developing, for detailed and accurate information about the interactions of GFNs at the molecular, cellular, and tissue levels, their physicochemical properties as well as their *in vitro* and *in vivo* ocular toxicities must be evaluated. Therefore, GFNs’ biocompatibility, stability, and biological performances as well as their side effects in ocular applications can be preliminarily obtained by considering these items. However, future research is necessary for exploring the biological responses and the safety issues of GFNs by taking into consideration the different physicochemical properties. Before doing any research, personal safety protection is crucially needed when dealing with GFNs both in production and in a research environment. All of these provided results will further improve the required knowledge for developing safe technologies and products using GFNs appropriate for biomedical applications and minimizing the risks to human health. Consequently, further research is still necessary to overcome the aggregation problems and genotoxicity of GFNs as well as their toxicity dependency on the size- and/or functional groups before we can draw a conclusion whether GFNs are safe or not.

## Abbreviations

GFNs: graphene family nanomaterials
CNT: carbon nanotube
0D: zero-dimensional
FLG: few-layer graphene
GO: graphene oxide
rGO: reduced graphene oxide
GQDs: graphene quantum dots
ROS: reactive oxygen species
hMSCs: human mesenchymal stem cells, LOG, low oxidized graphene
HOG: higher oxidized graphene
PEG: poly(ethylene glycol)
PEG-GO: PEGylated GO
PLL: PEGylated poly-l-lysine
CNMs: carbon-based nanomaterials
RPE: retinal pigment epithelium
MDA: malondialdehyde
Ti: titanium
TCPS: tissue culture plastic surface
LDH: lactate dehydrogenase
IOP: intraocular pressure
G-OH: hydroxylated graphene
hCorECs: human corneal epithelium cells
hConECs: human conjunctiva epithelium cells
GSH: glutathione
rGOM: rGO membrane
CPH: ciprofloxacin HCl
HA: hyaluronic acid
DOX: doxorubicin
Tf: transferrin
HTCC: including quaternized chitosan
Vor: voriconazole
BCEs: bioinspired compound eyes
IOP: intraocular pressure
FOV: fields of view
G/GNSs: glycerol/graphene nanosheets
nIR: near-infrared
EOG: electrooculography
ERG: electroretinography
PDMS: polydimethyl siloxane
GNWs: graphene nanowalls
GWFs: graphene woven fabrics
